# Clinicopathological Profile of Non-small Cell Lung Cancer and the Changing Trends in Its Histopathology: Experience From a Tertiary Care Cancer Center in Kashmir, India

**DOI:** 10.7759/cureus.34120

**Published:** 2023-01-23

**Authors:** Mohmad H Mir, Farhana Siraj, Nazia Mehfooz, Mushtaq A Sofi, Nisar A Syed, Nazir A Dar, Naseer A Choh, Sumyra K Qadri, Gull M Bhat

**Affiliations:** 1 Department of Medical Oncology, Sher-I-Kashmir Institute Of Medical Sciences, Srinagar, IND; 2 Department of General Medicine, Sher-I-Kashmir Institute of Medical Sciences, Srinagar, IND; 3 Department of Pulmonary Medicine, Sher-I-Kashmir Institute of Medical Sciences, Srinagar, IND; 4 Department of Radiation Oncology, Sher-I-Kashmir Institute of Medical Sciences, Srinagar, IND; 5 Department of Medical Oncology, Sher-I-Kashmir Institute of Medical Sciences, Srinagar, IND; 6 Department of Radiology, Sher-I-Kashmir Institute of Medical Sciences, Srinagar, IND; 7 Department of Pathology, Sher-I-Kashmir Institute of Medical Sciences, Srinagar, IND

**Keywords:** ecog performance status, kashmir, squamous cell carcinoma, adenocarcinoma, hookah smoking, non-small cell lung cancer

## Abstract

Background

The overall frequency and incidence of different cancers across the globe, including lung cancer, are marked by ethnic and geographical variations. Lung cancer is the most commonly diagnosed cancer worldwide that inflicts most of the cancer deaths. Non-small cell lung cancer (NSCLC) constitutes most lung cancer cases. The aim of this study was to find the frequency and clinicopathological characteristics of NSCLC in high incidence zone of Kashmir, an ethnically and geographically distinct area in Northern India.

Material and methods

The study was conducted to evaluate the clinicopathological profile of NSCLC at a tertiary care cancer center, Sher-I-Kashmir Institute of Medical Sciences (SKIMS). The patients and case records were analyzed for clinical presentation and demographic features, smoking status, radiological features, histopathological type, and stage of their disease at presentation.

Results

The study included 1557 NSCLC patients registered over a period of seven years, i.e., 2008-2014. Most of the patients belonged to rural areas (70%). The median age of the whole cohort was 58.0 years (ranges 22-95 years), and the male-to-female ratio was 3.7:1 (male = 1231 and female = 326). Smokers comprised 77.39% of cases, and Hookah was the most common form of smoking (65.06%). The ratio of squamous cell carcinoma and adenocarcinoma was 3.7:1 (67.5% vs. 24.9%). Stage III and IV disease accounted for 93% of cases (30.6% and 62.7%, respectively). Squamous cell carcinoma histopathology was dominant in smokers (74.3%) compared to adenocarcinoma (19%), while squamous cell carcinoma and adenocarcinoma histopathology ran parallel in nonsmokers (45.1% and 44%, respectively). Most of the patients had an Eastern Cooperative Oncology Group (ECOG) performance status between 1 and 2 (79%).

Conclusion

We conclude Kashmir region is a very high-risk area for lung cancer, with NSCLC showing a high incidence. Most of our patients present in advanced stages, and the frequency of adenocarcinoma is showing an increasing trend over the years from 2008-2014.

## Introduction

Globally, lung cancer is the major cause of cancer-related deaths and accounts for 18% of all cancer-related deaths. Combined, it is the second most common cancer occurring after breast cancer and the most common cancer (14.3%) in men [1]. In India, lung cancer accounts for 5.9% of all cancers and 8.1% of all cancer-related deaths [2]. In Kashmir, the landlocked valley in north India, lung cancer is a major health issue, ranks at the top among the 10 leading cancers, and constitutes 10.6% of all cancers [3]. Lung cancer is historically divided into two broad groups: small-cell lung cancer (SCLC) and non-small-cell lung cancer (NSCLC). NSCLC comprises approximately 85% of all primary lung cancers [4]. The major subtypes of non-small cell lung cancer (NSCLC) are adenocarcinoma (40%), which is the most common subtype, followed by squamous cell carcinoma (25%), and large cell carcinoma (10%) [5]. The pattern of lung cancer has been changing in the Western world. Lung cancer is becoming increasingly common in women [6]. Adenocarcinoma histopathology has surpassed squamous cell carcinoma as the most common type in both men and women [7].

Lung cancer occurrence and mortality are closely associated with the pattern of cigarette smoking. With the increase in the frequency of smoking rates, lung cancer has shown an increase in both incidence and mortality until recent years, with the widespread tobacco control programs helping to reduce its occurrence [8]. Smoking is an important risk factor for the development of lung cancer. Kashmir, a part of the Union Territory of Jammu & Kashmir in Northern India, is a mountain-locked region, and the number of cancer cases there is rising with every passing year. The region presents a diverse pattern and distribution of the cancer spectrum compared to most parts of the world, including the rest of India. The uniqueness of the diverse distribution of cancer in Kashmir is probably attributed to the different dietary habits and unique geographical position. The vast majority of lung cancers, approximately (85%) develop in the lifetime of (former or current) smokers. Like the rest of the world, cigarette smoking is common in this part of India as well, but in addition, hookah smoking, an indigenous form of smoking, is highly prevalent and largely responsible for the passive smoking of other family members, in particular during the winter [9-12]. In this regard, we conducted this study to examine the clinical and pathological profile of non-small cell lung cancer and evaluate the changing histopathological patterns of this cancer in Kashmir valley.

## Materials and methods

This hospital-based observational study was conducted in the department of medical oncology in the Regional Cancer Center at Sher-I-Kashmir Institute of Medical Sciences (SKIMS). The study was reviewed and approved by the institutional ethics committee. All histopathologically documented NSCLC patients registered at Regional Cancer Center (RCC) SKIMS were included. The data was collected with due care to include all the relevant information. All the patients included in the study were assessed clinically with history and complaints of illness, demography, and detailed documentation of clinical examination was performed. For documentation of performance status (PS), the Eastern Cooperative Oncology Group (ECOG) performance status score was used. Pre-treatment evaluation included a complete blood count, complete serum chemistry, chest X-ray, electrocardiogram (ECG), bronchoscopy, contrast-enhanced computed tomography (CECT) of the chest and upper abdomen, positron emission tomography (PET)/CT and brain imaging with contrast-enhanced magnetic resonance imaging (CE-MRI) as needed, a bone scan, histopathology of the lung lesions, fine-needle aspiration cytology (FNAC) of any peripheral node or any swelling if present, analysis of pleural effusion, and other necessary investigations as deemed necessary. Histopathology was done by the same group of pathologists. In the retrospective arm (2008-2012), the clinical and demographic information was collected from the case record files and entered in a predesigned proforma. Using the above investigations, a proper tumor-node-metastasis (TNM) stage of the disease was determined as per the American Joint Committee on Cancer (AJCC) seventh edition.

Ethics

This study was carried out in accordance with the institutional ethics committees (IEC) of SKIMS and the updated Helsinki Declaration from 1964. The study's approval was granted by the IEC of SKIMS in accordance with protocol RP-162/2014.

Data analysis

Descriptive statistics were used to describe demographic and clinical characteristics. All the continuous variables were shown in terms of frequency and percentages. Chi-squared test was used at appropriate places. SPSS version 24 (IBM Inc., Armonk, New York) was used for data analysis.

## Results

A total of 1557 histopathologically confirmed cases of NSCLC were registered during the study period. The median age of patients was 58 years (range: 22-95 years). Most of the patients in our study were in the age range of 50 to 70 years (60.3%). 19.07% belonged to the 40 to 50 years age group. The majority of our patients belonged to rural areas, n = 1090 (70.0%), as the city of Srinagar is the only urban habitat in the Kashmir Valley that accounted for most NSCLC cases, n = 467 (30.0%). The detailed demographics are listed in Table 1.

**Table 1:** Demography of studied NSCLC patients (n = 1557) NSCLC = non-small cell lung cancer; SCC = squamous cell carcinoma; ADC = adenocarcinoma; LCC = large cell carcinoma; ACC = adenoid cystic carcinoma; CECT = contrast-enhanced computed tomography; PET-CT = positron emission tomography-computed tomography; ECOG - Eastern Cooperative Oncology Group; PS - performance score *poorly differentiated, adenosquamous, NOS, etc.

Variable		Number	Percentage (%)
Age (years)	21-30	25	1.61
	31-40	110	7.06
	41-50	297	19.07
	51-60	528	33.91
	61-70	411	26.4
	71-80	168	10.79
	>80	18	1.16
Gender	Male	1,231	79.06
	Female	326	20.94
Dwelling	Rural	1,090	70
	Urban	467	30
Smoking status	Smoker	1,205	77.39
	Hookah	784	65.06
	Cigarette	311	25.81
	Mixed	110	9.13
	Nonsmoker	352	22.61
Histopathology	SCC	1,051	67.5
	ADC	389	24.98
	LCC	30	1.93
	ACC	11	0.71
	Others*	76	4.88
Stage	Stage IA	5	0.32
	Stage IB	7	0.45
	Stage II	47	3.02
	Stage IIIA	156	10.02
	Stage IIIB	320	20.55
	Stage IV	976	62.68
	Full staging not available	46	2.95
Radiological findings (CECT chest/PET-CT)	Right lung lesion	967	62.11
	Left lung lesion	464	29.8
	Bilateral lung lesions	45	2.89
	Pleural effusion	254	16.31
	Mass lesion with locoregional lymph nodes	579	37.19
	Collapse/consolidation	514	33.01
	Combined findings	239	15.35
ECOG PS score	1	528	33.91
	2	718	46.11
	3	82	5.26
	4	57	3.66
	Not available	172	11.04
Site of metastasis	Bones	247	25.3
	Pleura with positive pleural effusion	185	18.95
	Brain	154	15.77
	Multiple sites of metastasis	72	7.37

Males comprised 1,231 cases (79.06%) as against 326 (20.94%) females, and the male-to-female ratio of NSCLC patients was 3.7:1. Apart from cough with or without sputum production in 72.19% of cases, the other common presenting symptoms were breathlessness (31.79%), hemoptysis (14.90%), chest pain (13.55%), and weight loss (11.50%). Two patients with adenocarcinoma presented with the rare symptom of vision loss and, on evaluation, were found to have choroidal metastasis. Two patients presented with skin and subcutaneous metastases as their primary presentations. 77.39% of patients were smokers, compared to 22.61% of nonsmokers. Hookah smoking (Jajeer in local culture) in both males and females (65.06%) was the most common form of smoking in our study, as shown in Table 1. Squamous cell carcinoma was the most common histopathological sub-type (67.50%), followed by adenocarcinoma (24.98%). Large cell carcinoma was seen in 1.92%, adenoid cystic carcinoma in <1%, and others, which included poorly differentiated carcinoma, adenosquamous cell carcinoma, sarcomatoid carcinoma, and carcinoma not otherwise specified aggregated to 4.88%. Smoking was associated with all histological subtypes. The association of smoking with various histologies was significant compared to nonsmokers (p < 0.001). In all histological types, smoking was more common in males (81.96%) compared to females (60.12%). 

The majority of our patients had an ECOG performance status score between 1 and 2 (80%). We found that 9% of patients had an ECOG performance status score of 3 or 4. The majority of our patients had stage IV disease (62.68%). Stage I was present in <1%, stage II in 3.02%, stage IIIA in 10.02%, and stage IIIB in 20.55%. In the present study, the bones were the most common site of metastasis in NSCLC (25.30%), followed by the pleura with effusion (18.95%) and the brain (15.77%). Approximately 7.37% had combined sites of metastasis, as shown in Table 1 and Table 2

**Table 2:** Symptoms and signs of the studied patients (n = 1557)

Variable		N	%
Symptoms	Cough and sputum	1124	72.19
	Hemoptysis	232	14.9
	Breathlessness	495	31.79
	Chest pain	211	13.55
	Bone pains	63	4.05
	Hoarseness of voice	48	3.08
	Fever	55	3.53
	Neurological symptoms	37	2.38
	Weight loss	179	11.5
	Nodal swelling	19	1.22
	Skin and subcutaneous swelling and vision loss	4	0.26
Signs	Emaciated	93	5.97
	Pallor	319	20.48
	Clubbing	31	1.99
	Cyanosis	42	2.69
	Superior vena cava (SVC) obstruction/syndrome	29	1.86
	Lymphadenopathy	86	5.52
	Pleural effusion	188	12.07
	Signs of mass lesion	76	4.88
	Collapse/consolidation	84	5.39
	Hemiparesis/paraparesis	12	0.77
	Other (choroidal mass)	4	0.25

Tables 3, 4, 5 show NSCLC patients with their histological status in relation to varied parameters, like smoking and gender. In smokers, squamous cell carcinoma comprised 74.3% of cases, adenocarcinoma accounted for 19% of cases, and large cell carcinoma accounted for 1.8% of cases, while in nonsmokers, squamous cell carcinoma accounted for 44% and adenocarcinoma accounted for 45%. Among male smokers, 75.7% were squamous cell carcinoma cases vs. 18.9% adenocarcinoma cases, while for male nonsmokers, the frequency was 59% and 31%, respectively. For female smokers, 58.1% were squamous cell carcinoma cases as opposed to 39% adenocarcinoma, while nonsmokers had 34.6% squamous cell carcinoma and 49.6% cases of adenocarcinoma. Most of the patients presented with advanced-stage of disease 10.02%, 20.55%, and 62.68% with stage IIIA, stage IIIB, and stage IV, respectively, as shown in Table 6.

**Table 3:** Overall smoking status SCC = squamous cell carcinoma; ADC = adenocarcinoma; LCC = large cell carcinoma ACC = adenoid cystic carcinoma *poorly differentiated, adenosquamous, NOS, etc.

Variables	N	SCC	ADC	LCC	ACC	*Others
Smokers	1205	896	230	22	7	50
Nonsmokers	352	155	159	8	4	26
Total	1557	1051	389	30	11	76

**Table 4:** Gender-specific smoking status SCC = squamous cell carcinoma; ADC = adenocarcinoma; LCC = large cell carcinoma ACC = adenoid cystic carcinoma *poorly differentiated, adenosquamous, NOS, etc.

Variables	N	SCC	ADC	LCC	ACC	*Others
Male	1009	762	191	15	9	32
Female	196	114	66	4	0	12
Total	1205	876	257	19	9	44

**Table 5:** Gender-specific smoking status SCC = squamous cell carcinoma; ADC = adenocarcinoma; LCC = large cell carcinoma ACC = adenoid cystic carcinoma *poorly differentiated, adenosquamous, NOS, etc.

Variables	N	SCC	ADC	LCC	ACC	*Others
Male nonsmokers	222	131	69	8	1	13
Female nonsmokers	130	44	63	3	1	19
Total	352	175	132	11	2	32

**Table 6:** Stage of disease at presentation in studied patients

Stage of disease/year	2008	2009	2010	2011	2012	2013	2014 (till July)	Total	Percentage (%)
Stage IA	0	1	0	1	0	2	1	5	0.32
Stage IB	0	0	0	2	2	2	1	7	0.45
Stage II (IIA & IIB)	3	2	6	5	12	13	6	47	3.02
Stage IIIA	10	14	15	21	36	41	19	156	10.02
Stage IIIB	29	40	44	48	59	64	36	320	20.55
Stage IV	87	110	137	136	184	199	123	976	62.68
Full staging not available	5	4	6	5	8	13	5	46	2.95
Total	134	171	208	218	301	334	191	1557	100.00

## Discussion

Cancer ranks as a leading cause of death and an important barrier to increasing life expectancy in every country in the world [1]. With an estimated 2.2 million new cancer cases and 1.8 million deaths, lung cancer is the second most common cancer and the leading cause of cancer death in 2020, representing approximately one in 10 (11.4%) cancers diagnosed and one in five (18.0%) deaths. Lung cancer is the leading cause of cancer morbidity and mortality in men, whereas, in women, it ranks third for incidence, after breast and colorectal cancer, and second for mortality, after breast cancer. Incidence and mortality rates are roughly two times higher in men than in women [2]. Lung cancer has the highest incidence in developing nations where cigarette smoking is most prevalent, and it is most common in Russia, China, and much of Eastern Europe, the Middle East, and Southeast Asia. Among women, North America and northern and western Europe have the highest incidence worldwide. Western, central, and eastern Africa have the lowest incidence among men and women [4]. The valley of Kashmir, situated at an altitude of about 1,800 to 2,400 meters above sea level in the Indian Himalayas with a population of 7.0 million, is relatively isolated geographically, where consanguineous marriage is very common. Traditionally, the region has its own specific cultural norms, customs, and different dietary habits. The question arises whether lung cancer is following a similar trend in incidence and pattern as compared to the rest of the world. In various hospital-based studies conducted earlier in this region, lung cancer was rated as the second or third most common cancer in males, with around 80% to 85% comprising the non-small cell type [5-7]. The pattern of lung cancer has been changing in the Western world. Lung cancer is becoming increasingly common in women [6]. Adenocarcinoma histology has surpassed squamous cell carcinoma as the most common type in both men and women [7]. In 2021, lung cancer constituted 10.6% of total cancer cases [3]. In our study, we found an incidence of 6.55 and 1.88 cases per 100,000 per year of lung cancer in males and females, respectively, which contributes to nearly 0.004 cases of the total incidence of 1,825 cases in the world. For India, with an overall incidence of 71 cases per 100,000 per year, our region contributes hugely, with nearly 10.7% of cases of lung cancer [5, 8].

India still seems to have a lower incidence in the global race, as shown in Table 7, and the reason can be low reporting and diagnosis of the cases, in particular in rural areas. An earlier five-year study from our hospital on the profile of lung cancer had 321 patients with lung cancer, of whom 55 cases (17.1%) were of the small cell type, and the rest were NSCLC (n = 266; 82.9%), with the majority being squamous cell lung cancers (n = 248; 93.2%), and adenocarcinomas constituted 6.4%. Thus, the adenocarcinoma histological subtype of NSCLC is increasing year over year, and it was the second most common type of NSCLC in our current study (24.90%). Overall, squamous cell carcinoma was common in both sexes. The data presented here differs from earlier studies from the same region that had shown NSCLC constituting 80% to 85%, with squamous cell type being most common (67.5-77.3%) and adenocarcinoma being the least common (3-5.3% vs. 24.9% in our study) [5,7,8]. One of the reasons for the increase in adenocarcinoma at our center could be related to the overall increase in evaluation and reporting at our oncology center, and the increasing trend may also be attributed to some idiopathic changes. Further, the steady increase in the incidence of adenocarcinoma could be somewhat explained by a consistent increase in tobacco smoking. Multiple studies have found a dose-response relation between adenocarcinoma and cigarette smoking as compared with squamous cell carcinoma [13,14]. In addition, the relative risk for adenocarcinoma decreases more slowly after smoking cessation than that for squamous cell carcinoma [15]. Our region has been observing a constant increase in cigarette smoking and its expenditure, which has elevated overall NSCLC incidence, including adenocarcinoma [8].

**Table 7:** Estimated incidence of lung cancer per 100,000 population

Geographical region	Point incidence /100,000	Male	Female
World	1825.0	1242.0	583.0
USA	214.0	112.0	102.0
European Union (EU-28)	313.0	214.0	99.0
China	652.0	459.0	193.0
India	71.0	54.0	17.0
Kashmir		6.55	1.18

In radiology studies, 52% to 75% of adenocarcinomas present as peripheral nodules [16-18]. This may explain why there might be an underestimate of the proportion of adenocarcinomas in the bronchoscopy series. However, the increased use of flexible bronchoscopy has facilitated access to the lung periphery, and this may have led to an increase in the reported proportion of lung adenocarcinomas.

Looking at international data, adenocarcinoma has surpassed squamous cell carcinoma, as substantiated by a series of studies [19, 20]. In India, in some places, adenocarcinoma of the lung is the most common type, but many centers still show squamous cell carcinoma as the most common one [21-23].

The median age of our patients in the current study was 58.0 years, which is comparable to other Indian studies [24,25]. It is a decade younger than in the West [26], possibly because the population in the West is aging in general, and longevity is greater. Most of the patients in our study were males, a common trend [21,22,25] with a male-to-female ratio of 3.7:1, as compared to 11.3:1 in an earlier study from the same region. Smoking was common in our patients, with 77.39% smokers. Hookah was the most common mode of smoking in our patients, which is substantiated by other studies as well [10]. Hookah smoking is associated with a significantly higher risk for lung cancer in the Kashmiri population, with about a six-fold elevated risk as compared to nonsmoking controls [10]. In various Indian studies, the association of smoking with lung cancer varies between 47% and 91%, and most series report bidi smoking, an indigenous form of smoking, as the most common form of smoking in India [27]. Its causal relation with smoking status is already confirmed in multiple studies across the globe where a dose-response relationship has been established with the frequency of cigarettes smoked per day and the risk of lung cancer development. In our study, smoking was more common in men than in women. Most of the other studies also showed smoking associated with lung cancer more in males as compared to females [23-25]. In agreement with other studies, we found right lung lesions and mass lesions to be more common [25]. Most of the patients in our study had an ECOG performance status score of 1 or 2. Although advanced cancer was seen in the majority of our patients, they still had a lower ECOG score, again pointing to the indolent nature of lung cancer and the need for screening. The appearance of symptoms seems to be delayed compared to the appearance of cancer, so valuable time can be saved by early screening. In our study, pleural fluid was positive for malignant cells in 3.91% of cases, which is higher compared to that seen in another study (0.99%) [19]. Around 83% of our patients had locally advanced or stage IV disease (IIIB and IV), as agreed in other studies (71.8-73.29%) for stage IV at diagnosis [23,25]. In our study, the most common site of metastasis was the bone, while in other studies, the brain was the common site of metastasis [20,28]. The results are contrary to our study, where we had more cases of bone metastasis, probably because there were more cases of squamous cell carcinoma in our study.

## Conclusions

Lung cancer is the most frequently diagnosed cancer and is one of the most frequent causes of cancer-related deaths in both men and women in many countries of the world. NSCLC comprises the bulk of lung cancer cases. The incidence of lung cancer shows an increasing trend in the Kashmiri population, and it is currently the most commonly registered cancer at our center. Globally adenocarcinoma histology forms the major subtype of NSCLC. The proportion of adenocarcinoma has shown an increase in incidence, however squamous cell carcinoma is still the major subtype of NSCLC in our region, which is in contrast to the world data, and we hypothesize that it may be related to the high incidence of indigenous hookah smoking prevalent in our society. Most of our patients present with locally advanced and/or metastatic stages of the disease and are often offered palliative treatment. We believe low-dose CT screening should be implemented in high-risk patients for lung cancer in our region, and we suggest strong legislation from the law enforcing authorities for banning smoking in any form.

## References

[1] Sung H, Ferlay J, Siegel RL, Laversanne M, Soerjomataram I, Jemal A, Bray F (2021). Global cancer statistics 2020: GLOBOCAN estimates of incidence and mortality worldwide for 36 cancers in 185 countries. CA Cancer J Clin.

[2] Bray F, Ferlay J, Soerjomataram I, Siegel RL, Torre LA, Jemal A (2018). Global cancer statistics 2018: GLOBOCAN estimates of incidence and mortality worldwide for 36 cancers in 185 countries. CA Cancer J Clin.

[3] Khan NA, Syed NA, Dar NA, Masoodi SR, Lone MM (2021). Changing pattern of common cancers in last 5 years in Kashmir, India: a retrospective observational study. Indian J Med Paediatr Oncol.

[4] Sher T, Dy GK, Adjei AA (2008). Small cell lung cancer. Mayo Clin Proc.

[5] Travis WD, Brambilla E, Nicholson AG (2015). The 2015 World Health Organization Classification of lung tumors: impact of genetic, clinical and radiologic advances since the 2004 classification. J Thorac Oncol.

[6] Maule M, Merletti F (2012). Cancer transition and priorities for cancer control. Lancet Oncol.

[7] Siegel RL, Miller KD, Jemal A (2018). Cancer statistics, 2018. CA Cancer J Clin.

[8] Rosenow EC (1993). Symposium on intrathoracic neoplasm. Mayo Clin Proc.

[9] Koul PA, Kaul SK, Sheikh MM, Tasleem RA, Shah A (2010). Lung cancer in the Kashmir valley. Lung India.

[10] Thun MJ, DeLancey JO, Center MM, Jemal A, Ward EM (2010). The global burden of cancer: priorities for prevention. Carcinogenesis.

[11] Pandith AA, Siddiqi MA (2012). Burden of cancers in the valley of Kashmir: 5 year epidemiological study reveals a different scenario. Tumour Biol.

[12] Khan NA, Afroz F, Lone MM, Teli MA, Muzaffar M, Jan N (2006). Profile of lung cancer in Kashmir, India: a five-year study. Indian J Chest Dis Allied Sci.

[13] Brownson RC, Reif JS, Keefe TJ, Ferguson SW, Pritzl JA (1987). Risk factors for adenocarcinoma of the lung. Am J Epidemiol.

[14] Higgins IT, Wynder EL (1988). Reduction in risk of lung cancer among ex-smokers with particular reference to histologic type. Cancer.

[15] GK Web Desk: Health, Srinagar Srinagar (2016). Smokers spend more on cigarettes in Jammu Kashmir than rest of India. https://www.greaterkashmir.com/news/life-style/smokers-spend-more-on-cigarettes-in-jammu-kashmir-than-rest-of-india/217354.html.

[16] Theros EG (1977). 1976 Caldwell Lecture: varying manifestation of peripheral pulmonary neoplasms: a radiologic-pathologic correlative study. AJR Am J Roentgenol.

[17] Woodring JH, Stelling CB (1983). Adenocarcinoma of the lung: a tumor with a changing pleomorphic character. AJR Am J Roentgenol.

[18] Arrieta O, Campos-Parra AD, Zuloaga C (2012). Clinical and pathological characteristics, outcome and mutational profiles regarding non-small-cell lung cancer related to wood-smoke exposure. J Thorac Oncol.

[19] Houston KA, Henley SJ, Li J, White MC, Richards TB (2014). Patterns in lung cancer incidence rates and trends by histologic type in the United States, 2004-2009. Lung Cancer.

[20] Noronha V, Dikshit R, Raut N (2012). Epidemiology of lung cancer in India: focus on the differences between non-smokers and smokers: a single-centre experience. Indian J Cancer.

[21] Malik PS, Sharma MC, Mohanti BK (2013). Clinico-pathological profile of lung cancer at AIIMS: a changing paradigm in India. Asian Pac J Cancer Prev.

[22] Rawat J, Sindhwani G, Gaur D, Dua R, Saini S (2009). Clinico-pathological profile of lung cancer in Uttarakhand. Lung India.

[23] Bhattacharyya SK, Mandal A, Deoghuria D, Agarwala A, Ghoshal AG, Dey SK (2011). Clinico-pathological profile of lung cancer in a tertiary medical centre in India: analysis of 266 cases. J Dent Oral Hyg.

[24] Behera D, Balamugesh T (2004). Lung cancer in India. Indian J Chest Dis Allied Sci.

[25] Jemal A, Murray T, Samuels A, Ghafoor A, Ward E, Thun MJ (2003). Cancer statistics, 2003. CA Cancer J Clin.

[26] Bhaskarapillai B, Kumar SS, Balasubramanian S (2012). Lung cancer in Malabar Cancer Center in Kerala - a descriptive analysis. Asian Pac J Cancer Prev.

[27] RA J, FA B (1962). Study of narghile smoking in relation to cancer of the lung. Br J Cancer.

[28] Quint LE, Tummala S, Brisson LJ (1996). Distribution of distant metastases from newly diagnosed non-small cell lung cancer. Ann Thorac Surg.

